# Medroxyprogestogen enhances apoptosis of SKOV-3 cells via inhibition of the PI3K/Akt signaling pathway

**DOI:** 10.7555/JBR.27.20120051

**Published:** 2012-12-10

**Authors:** Yan Li, Yi Jiang, Yicong Wan, Lin Zhang, Weiwei Tang, Jingjing Ma, Shan Wu, Wenjun Cheng

**Affiliations:** a Department of Gynecology and Obstetrics, the First Clinical Medical College, Nanjing Medical University, Nanjing, Jiangsu 210029, China;; b Department of Gynecology, the First Affiliated Hospital, Nanjing Medical University, Nanjing, Jiangsu 210029, China.

**Keywords:** medroxyprogestogen, ovarian cancer, Akt, phosphorylation, apoptosis

## Abstract

We sought to assess the effect of progestin on the apoptosis of epithelial ovarian cancer cell line SKOV-3 and via regulation of phosphorylation signaling in. Epithelial ovarian cancer cell line SKOV-3 was treated with medroxyprogestogen, phosphatidylinositol 3-kinase inhibitor LY294002 and vehicle control. Akt, phospho-Akt, Bcl-2 and phospho-Bad proteins were examined by immunoblotting assays. Medroxyprogestogen-induced apoptosis was assessed by MTT assays and Annexin V apoptosis assay. We found no significant difference in Akt and Bad expression in both the medroxyprogestogen groups and the control group. The levels of phospho-Akt, Bcl-2 and phospho-Bad were decreased in all the medroxyprogestogen groups and significantly decreased in the high dose mitogen-activated protein (MAP) group (10 µmol/L). Viability of SKOV-3 was reduced and apparent apoptosis of SKOV-3 cells was observed with increased doses of MAP. The findings suggest that medroxyprogestogen can induce SKOV-3 cell apoptosis by inhibiting Akt phosphorylation.

## INTRODUCTION

Ovarian carcinoma is the second most common and the most deadly malignancy of the female reproductive system[1]. The molecular mechanisms of ovarian oncogenesis are poorly understood. A previous study revealed that while postmenopausal estrogen replacement therapy using non-progestin regimens was a risk factor of carcinogenesis in ovarian cancer, hormone replacement therapy (HRT) with progestin did not increase the incidence of ovarian cancer[2]. Instead, the use of combination oral contraceptives (estrogen combined with progestin) is associated with a decrease in the incidence of ovarian cancer[3],[4]. Furthermore, a high dose of progestin reduces the risk of ovarian cancer to a greater extent than low-dose progestin formulations in women[5], suggesting a protective effect of progesterone against ovarian carcinogenesis. However, the underlying mechanisms for the correlation between the use of progesterone and ovarian carcinogenesis remain unclear.

We attempt to determine the mechanism of progestin-induced apoptosis in ovarian cancer cells. SKOV-3 cells were treated with serial concentrations of mitogen-activated protein (MAP), and some of the Bcl-2 family proteins were detected in dose-dependent experiments. Bcl-2 family proteins are known to determine the outcome of an intrinsic apoptotic process initiated by the release of cytochrome C and apoptotic factors from the mitochondria[6]. Bcl-2-associated death promoter (Bad), a BH3-only protein, is one of the “death-promoting” members of the Bcl-2 family and its pro-apoptotic activity is regulated primarily by phosphorylation at several sites[7],[8]. Survival factors induce Bad phosphorylation via several protein kinase signaling pathways including the activation of the phosphatidylinositol 3-kinase (PI3K)-Akt[9],[10] pathway and the mitogen-activated protein kinase (MAPK)-ribosomal S6 kinase (RSK) pathway[11],[12]. Phosphorylated Bad associates with 14-3-3 proteins in the cytoplasm, preventing translocation of Bad to the mitochondria[13] and interaction with the anti-apoptotic proteins Bcl-2 and Bcl-xl[8],[14]. These proteins, freed from Bad, in turn associate with two other pro-apoptotic proteins, Bax and Bak. Such association prevents aggregation of these pro-apoptotic proteins on the mitochondrial membrane, suppressing cytochrome C release and consequently inhibiting apoptosis[6],[14].

As we all know that among cell growth and survival controlling mechanisms, the PI3K signaling pathway is often activated. Numerous reports suggested that PI3K plays a role in signaling in invasion and metastasis[15]-[20] in various kinds of carcinomas. It is shown that Akt was amplified or overexpressed in ovarian cancer, implying that it also plays a role in ovarian oncogenesis[21]. Abundant evidence indicated that progestin opposes the effect of estrogen in tumorigenesis. Indeed, several progestogens have been shown to repress the PI3K-Akt pathway[22].

In the present study, we used SKOV-3 ovarian cancer cell line as a model for ovarian cancer in vitro. Cell viability assays and annexin V/propidium iodide apoptosis assays were performed to assess the effect of progesterone in facilitating apoptosis. To ascertain the effect of progesterone in suppressing the PI3K pathway, we characterized the expression of Akt and phosphorylated Akt (phospho-Akt) after cells were treated with serial concentrations of MAP. Furthermore, Bcl-2 levels and Bad phosphorylation were examined to determine whether the PI3K-Akt-Bad signaling pathway played a role in regulating cell apoptosis and whether this mechanism was targeted by MAP in ovarian cancer cells.

## MATERIALS AND METHODS

### Materials

SKOV-3 cells were obtained from the Gynecology Department of Fudan University, Shanghai. Dulbecco's modified Eagle's medium (DMEM) and fetal bovine serum (FBS) were supplied by Gibco-BRL (Gran Island, NY, USA). MAP was purchased from Sigma-Aldrich (Gillingham, UK). Antibodies against Akt, phospho-Akt (Ser473), Bcl-2, phospho-Bad (Ser136) and β-actin were purchased from Santa Cruz Biotechnology (Santa Cruz, CA, USA). PI3K inhibitor LY294002 and the enhanced chemiluminescence (ECL) Western blotting detection reagents were supplied by Cell Signaling Technology (Beverly, MA, USA). Annexin V-FITC apoptosis detection kit was obtained from Biouniquer Technology Co., Ltd (Shanghai, China).

### Cell culture

SKOV-3 cells were maintained as monolayer cultures in minimal DMEM supplemented with 10% FBS and 1% antibiotics in a humidified chamber with 5% CO_2_ at 37°C.

### Treatment of SKOV-3 with MAP and PI3K kinase inhibitor

To determine the effect of MAP on cellular apoptosis and the expression of Akt, phospho-Akt, Bad, phospho-Bad and Bcl-2, we pretreated SKOV-3 cells with DMEM containing 10% charcoal-treated FBS for 72 hours. In dose-dependent experiments, Akt, phospho-Akt, Bad, phospho-Bad and Bcl-2 expression were investigated after MAP treatment, and cells were treated with serial concentrations (0.1, 1, 10, and 100 µmol/L) of MAP. To investigate the combined effect of MAP and pharmacological inhibitor of MAP, we treated SKOV-3 cells with 25 µmol/L LY294002 (IC50 1.4 µM) for 1 h before treatment with different concentrations of MAP. Control cells were treated with vehicle (DMSO, dimethyl sulfoxide) only. Apoptosis was evaluated by using MTT assays and an Annexin V Apoptosis Assay kit (Biouniquer Technology, Beijing, China). Akt, phospho-Akt, Bad, phospho-Bad and Bcl-2 were detected by Western blotting assays.

### Cell viability assay

Cell viability was determined calorimetrically using MTT reagents. Logarithmically growing SKOV-3 epithelial ovarian cancer cells were subcultured at 104 cells/well in 96-well microplates and incubated overnight. After different treatments, 20 µL of 5 mg/mL MTT solution was added to each well (0.1 mg/mL) and incubated for 4 h. The supernatants were aspirated and formazan crystals in each well were dissolved in 200 µL DMSO for 10 minutes at 37°C, and measured at 570 nm using an automatic multi-well spectrophotometer (Bio-Rad, Richmond, CA, USA). Each concentration included six wells and each experiment was repeated at least three times.

**Fig. 1:**
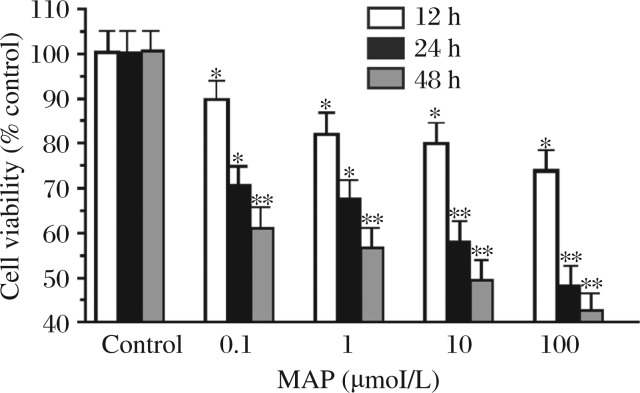
MAP suppresses SKOV-3 cell proliferation in vitro. Epithelial ovarian carcinoma cells (SKOV-3) were seeded into 96-well plates at 7,000 cells//well. Cell were incubated with serial concentrations (0.1, 1, 10, 100 µmol/L) MAP for 12, 24 and 48 hours. Cytostatic effects were measured using crystal violet staining and expressed as mean survival (as compared with controls) ±SD at least three independent experiments. The results suggest that MAP suppresses SKOV-3 cell viability in a dose- and time-dependent manner. *P < 0.05, **P < 0.01 (two-way ANOVA) in comparison with controls.

**Fig. 2:**
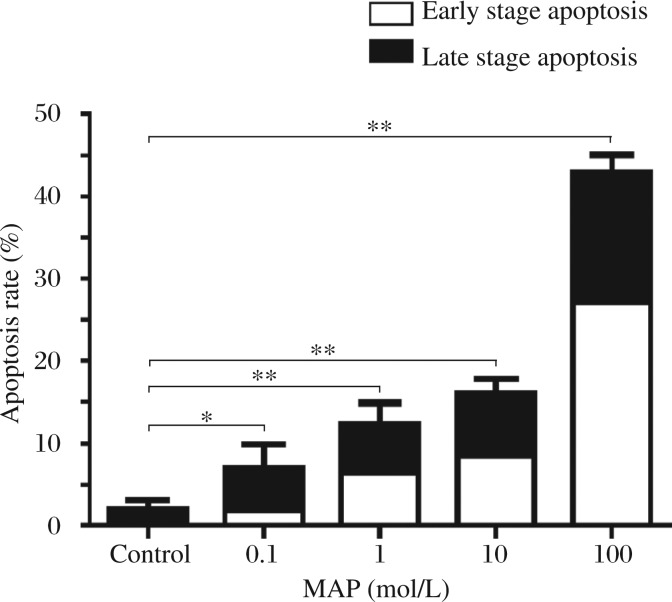
MAP induces apoptotic death of SKOV-3 cells Epithelial ovarian carcinoma cells (SKOV-3) were incubated with serial concentrations (0.1, 1, 10, 100 µmmol/L) MAP for 24 and 48 hours. Annexin V/propidium iodide apoptosis assay was used to measure apoptosis of SKOV-3. The results show that compared with the control group, the apoptosis in all MAP treated groups increased. Cells were treated with low dose MAP for 48 hours and the apoptosis rate increased to 24%. When cells were treated with high dose MAP, the apoptosis rate increased to 31% at 24 hours and 43% at 48 hours. *P < 0.05, **P < 0.01.

### Annexin V/propidium iodide apoptosis assay

SKOV-3 epithelial ovarian cancer cells were seeded onto 6-well plates and incubated overnight. The cells were divided into four groups and treated with 0.1, 1.0, 10, and 100 µmol/L MAP for 12, 24 and 48 hours at 37°C. Each group of cells (1×10^6^) were suspended in 500 µL binding buffer (10 mmol/L HEPES/NaOH, pH 7.4, 150 mmol/L NaCl, 5 mmol/L KCl, 1 mmol/L MgCl_2_, and 1.8 mmol/L CaCl_2_) and treated with 10 µL of FITC-conjugated anti-Annexin V-antibody and 10 µL of propidium iodide for 10 minutes in the dark at room temperature. The samples were analyzed by flow cytometry (excitation at 488 nm, and emission at 530 nm).

### Caspase-3 and -8 activity assays

Activation of caspase-3/caspase-8 activities was determined by caspase-3/caspase-8 assay kit, Colorimetric (Sigma Aldrich, St. Louis, MO, USA). Briefly, after sequential treatment of MAP, cells were lysed by lysis buffer (50 mmol/L HEPES, pH 7.4, 5 mmol/L CHAPS, and 5 mmol/L DTT). Protein concentration was determined by Bradford assay. The lysate containing 100 µg of protein in 90 µL assay buffer (20 mmol/L HEPES, pH 7.4, 2 mmol/L EDTA, 0.1% CHAPS, and 5 mmol/L DTT) were mixed with 10 µL caspase-3 or caspase-8 specific synthetic fluorescent substrates AC-DEVD-pNA/AC-IEVD-pNA (final concentration 200 µmol/L). After incubation at 37°C for 4 hours, yellowish color from the pNA released from the substrates by the action of active caspase-3/caspase-8 was determined at 405 nm on Fluorescence Reader (Bio-Tec Instruments Inc., Winooski, Vermont, USA)

### Gel electrophoresis and Western blotting

Cells were washed with cold PBS and harvested in RIPA buffer containing protease inhibitors. Cell lysates were incubated on ice for 30 minutes. After centrifugation at 25,000 *g* for 30 minutes at 4°C, protein concentrations were determined using a Bradford protein assay kit (Galen Biopharm International Co., Ltd, Beijing, China). Equal protein amounts were resolved by electrophoresis on 12.5% sodium dodecylsulfate polyacrylamide gels and then transferred to PVDF membranes with an SD Semi-dry Transfer Cell (Bio-Rad). The membranes were blocked with 5% evaporated skimmed milk in TBS for 2 hours at room temperature and then incubated overnight at 4°C with primary antibodies against the following primary proteins: Akt, phospho-Akt, Bcl-2, phospho-Bad, Bad, and β-actin. The blots were then incubated with horseradish peroxidase-conjugated anti-mouse IgG or anti-rabbit IgG for 60 minutes at room temperature and the signals were detected using ECL.

### Statistical analysis

Scanning densitometry was performed using an Image Master^®^ VSD (Pharmacia Biotech Inc., San Francisco, CA, USA). Significance test was performed using two-way analysis of variance (two-way ANOVA), and *P* values < 0.05 were accepted as significant.

## RESULTS

### MAP suppresses the viability of SKOV-3 cells

To determine the effect of MAP on ovarian cancer cell viability, SKOV-3 cells were treated with increasing concentrations (0.1-100 µmol/L) of MAP for 12, 24 and 48 h (***Fig. 1***). Incubation with high dose MAP (100 µmol/L) for 12 h and 48 h led to a 25% and 54% decrease in cell viability, respectively. While cell viability appeared unaltered at early time points in response to low dose MAP, treatment of SKOV-3 cells with MPA at a concentration as low as 0.1 µmol/L for a time period of 48 h led to an approximately 40% reduction in cell viability (***Fig. 1***). Thus, our data indicated that MAP suppressed SKOV-3 cell viability in a dose- and time-dependent manner.

### MAP induces apoptotic cell death in SKOV-3 cells

By using annexin V/propidium iodide apoptosis assays, we performed flow cytometry to confirm whether MAP could induce cellular apoptosis in a dose- (0.1, 1, 10 and 100 µmol/L) and time (24 and 48 hours) dependent manners. Compared with the control group, apoptosis in all MAP treated groups increased. As shown in ***Fig. 2***, cells were treated with low dose MAP for 48 h and the apoptotic rate increased to 24%. When cells were treated with high dose MAP, the apoptotic rate increased to 31% for 24 hours and 43% for 48 hours. The results suggested that MAP induced the apoptosis of SKOV-3 cells in dose-and-time dependent manners.

### MAP induces increased caspase-3 and -8 activities in SKOV-3 cells

**Fig. 3:**
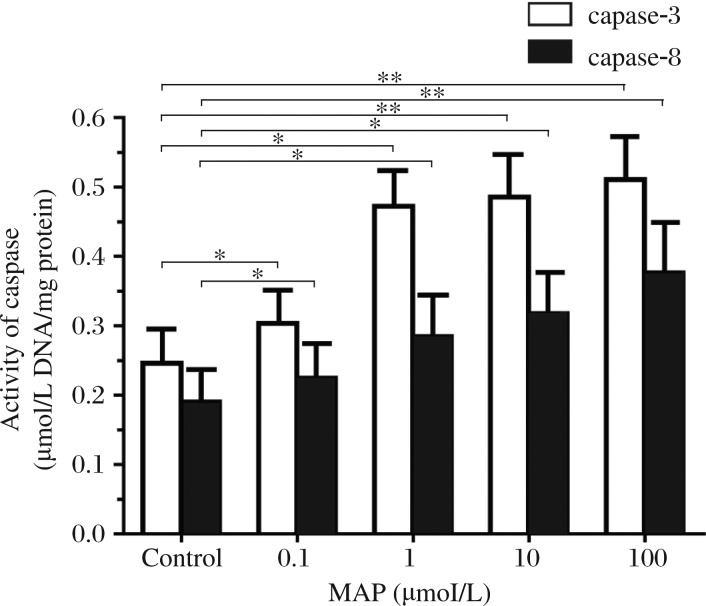
MAP induced-apoptosis is dependent on caspase-3 and caspase-8 activation. SKOV-3 cells were pretreated with different concentrations of MAP (0, 0.1, 1, 10, and 100 µmol/L) for 24 hours. The cells were harvested and lysed. The lysate containing 100 µMg of protein was mixed with caspase-3/caspase-8 specific synthetic fluorescent substrate AC-DEVD-pNA/ AC-IEVD-pNA. After incubation at 37°C for 4 hours or more, the yellowish color from the pNA released from the substrate by the action of active caspase-3 or caspase -8 were determined at 405 nm. Columns represent the mean (±SD) values obtained from three independent experiments. *P < 0.05, **P < 0.01.

Caspase-3 and -8 activities were detected after SKOV-3 cells were treated with serial concentrations of MAP for 24 hours. The results showed that MAP caused a dose-dependent increase in caspase-3 and -8 activities. When cells were treated with low dose MAP, caspase-3 activity was increased 60.1% and caspase-8 activity was increased 47.1%. When cells were treated with high dose MAP, caspase-3 activity was increased 82.6% and caspase-8 was increased 94.6% (***Fig. 3***).

**Fig. 4:**
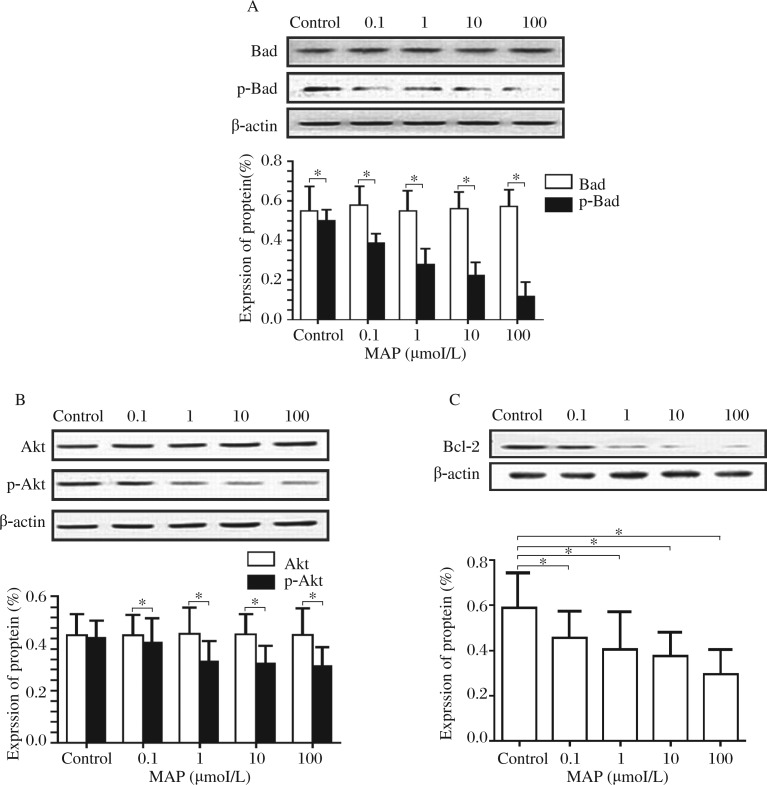
The expressions of Akt, p-Akt and Bad and p-Bad, and Bcl-2 proteins in SKOV-3. SKOV-3 cells were treated with serial concentrations of (0, 0.1, 1, 10, and 100 µmol/L) MAP for 24 hours. The protein levels of Akt and p-Akt (A), Bad and p-Bad (B), and Bcl-2 (C) were detected by Western blotting assays. Adobe Photoshop was used to measure the mean gray value of each band. The result suggested that exogenous MAP decreases p-Akt, Bcl-2 and p-Bad expressions in a dose-dependent manner, but causes no apparent changes in the expressions of Akt and Bad.

MAP induces apoptosis by inhibiting Akt and Bad phosphorylation and decreasing Bcl-2 expression in SKOV-3 cells. To determine whether MAP-induced cytotoxicity was due to induction of apoptotic cell death in SKOV-3 cells, we examined the levels of the apoptosis-associated proteins Bcl-2, Bad and phospho-Bad by Western blot analysis. To examine the relationship between Akt phosphorylation and cell apoptosis, we also determined the levels of Akt and phospho-Akt by Western blotting assays. SKOV-3 cells were treated with MAP at different concentrations for 4 hours to exam the levels of Akt, phospho-Akt, Bad, phospho-Bad and Bcl-2. Western blot analysis showed that MAP dose-dependently decreased the levels of phospho-Akt, phospho-Bad and Bcl-2, but caused no significant change in the protein level of Bad and Akt (***Fig. 4***). These results suggested that MAP-induced apoptotic cell death in SKOV-3 cells is likely through down-regulation of Akt and Bad phosphorylation and suppression of Bcl-2 expression.

### LY294002 attenuates MAP induced cell death

**Fig. 5:**
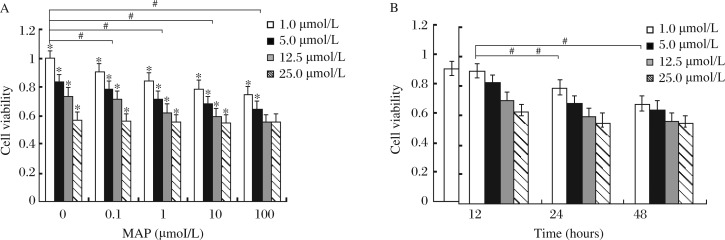
MAP and LY294002 induce epithelial ovarian carcinoma cell apoptosis in vitro. A: Epithelial ovarian carcinoma cells (SKOV-3) were seeded into 96-well plates at the concentration of 7,000 cells/well. Cell were treated with serial concentrations of LY294002 (1.0, 5, 12.5, and 25 µmol/L) for 1 hour. The cells were then incubated with serial concentrations (0.1, 1, 10, and 100 µmol/L) MAP for 24 hours. B: The cells were pretreated with LY294002 at the indicated doses. The cells were then incubated with 10 µmol/L MAP for 12, 24 and 48 hours. Cytostatic effects were measured using crystal violet staining and expressed as mean survival (as compared with controls) ±SD. Two-way analysis of variance was used to compare the measurement data between groups (^#^*P* < 0.05), while *t* test for comparison within groups (*P < 0.05).

In order to determine the role of the PI3K/Akt pathway, we pretreated SKOV-3 cells with the PI3K kinase inhibitor LY294002 (1, 5, 12.5, and 25 µmol/L) for 1 hour before the cells were treated with different concentrations of MAP for additional 12, 24, and 48 hours (***Fig. 5***). The results suggested that cell viability was gradually decreased in the same concentration of MAP with increasing concentrations of LY294002 (*P* < 0.05). Cell viability was gradually decreased in the same concentration of the LY294002 groups with increasing concentrations of MAP (the concentration of LY294002 was limited in 1-12.5 µmol/L, *P* < 0.05). Conversely, increasing of the concentration of MPA did not decrease cell viability when the concentration of LY294002 was 25 µmol/L.

## DISCUSSION

Standard treatment of advanced-stage ovarian carcinomas includes radical cytoreductive surgery, which aims to remove all visible tumor tissue followed by platinum and paclitaxel chemotherapy. Although ovarian cancer is chemosensitive, tumors eventually recur in two-thirds of patients, even after optimal surgical debulking followed by chemotherapy with platinum and taxane compounds. To improve clinical outcomes, combination of innocuous dietary components with anticancer drugs is an emerging new strategy for cancer chemotherapy to increase anticancer responses. In the present study, we found that MAP induced apoptosis in human ovarian cancer cells through targeting the PI3K/Akt signaling pathway. By inhibition of the PI3K-Akt pathway, the levels of Bcl-2 and Bad phosphorylation were decreased. Cell viability and annexin V/propidium iodide apoptosis assays also indicated that MAP suppresses SKOV-3 cell viability and induces apoptosis in a dose-and-time depended manner.

Progesterone has been tested as an adjunctive anticancer drug for long time. The interaction of progesterone and progesterone receptor (PR) in ovarian cancers has been focused on by a series of studies. One of these studies has shown that PR expression is a favorable prognostic factor for progression-free and overall survival[23],[24]. PR is an intracellular steroid receptor belonging to the nuclear receptor subfamily 3 (the group C, member 3) that specifically binds to progesterone. Upon progesterone binding, PR undergoes conformational changes and dimerization and the receptor-ligand complex then enters the nucleus and binds to DNA to regulate target gene transcription. Recent studies have shown that progesterone can also have a non-genomic cellular effect by acting in a PR-independent manner. Here, we found that treatment of the PR negative ovarian cancer cell line-SKOV-3 cells, with serial concentrations of MAP, led to reduced cell viability and apoptosis in a dose-and-time depended manner. These results are consistent with McDonnel's study, which showed that high-dose progesterone can inhibit urokinase secretion and invasive activity by SKOV-3 ovarian carcinoma cells, and this reaction was not altered by the progesterone receptor antagonist RU486 or the transcriptional inhibitor actinomycin D[25].

Given that MAP induced apoptosis in SKOV-3 cells, which lack functional PR, we sought to investigate the underlying mechanisms. The PI3K/Akt signaling pathway plays a significant role in cell growth, proliferation and tumorigenesis of various malignancies. Over the past years, substantial evidence has been accumulated regarding the therapeutic usefulness of PI3K pathway inhibitors for various malignancies including breast, gastrointestinal, head and neck, renal and other solid tumors[26]–[28]. We focused our attention on the functions of the PI3K/Akt pathway in cellular apoptosis and found that Akt phosphorylation, an indicator of PI3K/Akt pathway activation, was reduced in cells treated with MAP. It suggests that MAP induced inhibition of the PI3K/Akt pathway in these cells.

Bcl-2 family proteins are known to govern mitochondrial outer membrane permeability and be either pro-apoptotic or anti-apoptotic. Bcl-2 is the first protein identified to be involved in the regulation of apoptosis. Bcl-2 mediates anti-apoptotic effect by suppressing the production of reactive oxygen species by the mitochondrion and reducing the release of cytochrome c[29]. Bad protein is a pro-apoptotic member and a key effector downstream of the Bcl-2 family, which is involved in initiating apoptosis. Previous studies have shown that Akt can phosphorylate Bad and inhibit Bad-mediated apoptosis. In the present study, we also found that p-Bad and Bcl-2 levels were decreased and apoptosis was increased in SKOV-3 cells, suggesting that MAP may act to suppress cell viability through the PI3K-Akt-Bad pathway. These observations are consistent with previous studies showing that inhibition of the PI3K/Akt pathway reduced p-Bad and Bcl-2 expression and induced ovarian cancer cell apoptosis in vitro[22],[30]. Furthermore, after treatment of SKOV-3 cells with PI3K inhibitor LY294002, the functions of MAP were repressed. This result provides another piece of evidence that MAP can induce SKOV-3 cell apoptosis by reducing PI3K/Akt pathway activation.

The “canonical” function of progesterone via PR has been well documented. However, the PR-independent functions for progesterone to regulate apoptosis have not been well investigated. Our study investigated the relationship between progesterone induction of apoptosis and the PI3K/Akt pathway, which provided a new model to study hormone functions in ovarian carcinoma. High recurrence rate poses a serious threat to ovarian cancer patients. Effective and affordable treatments during chemotherapy-free intervals to reduce disease-relapse are currently lacking for ovarian cancer patients. Progesterone, as a natural sex hormone widely used for contraception with little side effect, has proved to be easily accessible and cost-effective. Indeed, progesterone has been used as an adjunctive therapy in chemotherapy-free intervals in estrogen and progesterone receptor-positive endometrial cancer for a long time. However, progestin therapy is not considered standard therapy for ovarian cancer so far despite ample evidence showing that progesterone can also be used as a chemotherapy drug sensitizer in ovarian cancer with beneficial effects both in vitro and in vivo. We found that progesterone used alone was sufficient to induce apoptosis in ovarian cancer cells, which do not express PR. Our results pointed to the potential usefulness of progesterone as a therapeutic agent for the treatment of ovarian cancer regardless of tumor PR status.
